# Metabolic Reprogramming of Cancer Associated Fibroblasts: The Slavery of Stromal Fibroblasts

**DOI:** 10.1155/2018/6075403

**Published:** 2018-06-05

**Authors:** Angelica Avagliano, Giuseppina Granato, Maria Rosaria Ruocco, Veronica Romano, Immacolata Belviso, Antonia Carfora, Stefania Montagnani, Alessandro Arcucci

**Affiliations:** ^1^Department of Public Health, University of Naples Federico II, 80131 Naples, Italy; ^2^Department of Molecular Medicine and Medical Biotechnology, University of Naples Federico II, 80131 Naples, Italy

## Abstract

Cancer associated fibroblasts (CAFs) are the main stromal cell type of solid tumour microenvironment and undergo an activation process associated with secretion of growth factors, cytokines, and paracrine interactions. One of the important features of solid tumours is the metabolic reprogramming that leads to changes of bioenergetics and biosynthesis in both tumour cells and CAFs. In particular, CAFs follow the evolution of tumour disease and acquire a catabolic phenotype: in tumour tissues, cancer cells and tumour microenvironment form a network where the crosstalk between cancer cells and CAFs is associated with cell metabolic reprogramming that contributes to CAFs activation, cancer growth, and progression and evasion from cancer therapies. In this regard, the study of CAFs metabolic reprogramming could contribute to better understand their activation process, the interaction between stroma, and cancer cells and could offer innovative tools for the development of new therapeutic strategies able to eradicate the protumorigenic activity of CAFs. Therefore, this review focuses on CAFs metabolic reprogramming associated with both differentiation process and cancer and stromal cells crosstalk. Finally, therapeutic responses and potential anticancer strategies targeting CAFs metabolic reprogramming are reviewed.

## 1. Introduction

In 1927, Warburg et al. reported that glucose metabolism was significantly enhanced in tumour cells compared with normal tissue, even in the presence of sufficient oxygen [1, 2]. This process known as “Warburg effect” is the principal and metabolic characteristic of cancer and is associated with metabolic reprogramming of cancer cells [3]. Moreover, other metabolic adaptations have been described in tumour tissues, such as the use of alternative carbon sources and the establishment of metabolic interactions between tumour and stromal cells represented by the “reverse Warburg effect” [3, 4]. Therefore, solid tumours can be described as metabolically heterogeneous diseases, in which several energetic pathways of tumour microenvironment collaborate [5, 6].

Furthermore, tumour microenvironment, including blood and lymphatic tumour vessels, extracellular matrix (ECM), and noncancer stromal cells such as cancer associated fibroblasts (CAFs), modulates cancer growth, progression, and evasion from cancer therapies [7]. In particular, CAFs are the major tumour stromal cells and are also prone, such as the cancer cells, to metabolic reprogramming leading to glycolysis switch [8]. Moreover, recent studies have showed the important role of CAFs in tumour initiation, progression, and metastasis [7, 9, 10].

From this point of view, the study of metabolic reprogramming that regulates CAFs differentiation and their crosstalk with cancer cells becomes a crucial topic in cancer research and could contribute to developing new therapeutic strategies destroying the protumorigenic activity of CAFs from cancer network [7, 8].

Therefore, in this review article, we summarized the role of metabolic reprogramming in CAFs differentiation and pointed out CAFs metabolic reprogramming mechanisms and cancer crosstalk.

Moreover, we discussed the significance of CAFs reprogramming mechanisms in cancer therapeutic responses and focused on the potential therapeutic strategies targeting molecules involved in CAFs reprogramming.

## 2. CAFs and Tumour Microenvironment Hallmarks

Fibroblasts represent a heterogeneous population of mesenchymal cells characterized by an exceptional phenotypic plasticity and capability to secrete large amounts of soluble factors, ECM components, and extracellular vesicles [11]. Under physiological conditions, fibroblasts regulate the turnover of ECM, control tissue homeostasis, and participate in wound healing and senescence [7]. On the other hand, in solid tumours, normal fibroblasts (NFs) differentiate to CAFs that coevolve with the disorder and alter the biochemical and physical structure of the tumour microenvironment, modifying the behaviour of the surrounding stromal and cancer cells [11, 12]. Therefore, CAFs are the most prominent noncancer cell type within the reactive stroma of many solid tumours [13] and are often described as cells in a constitutively activated state, sharing similarities with activated fibroblasts, named myofibroblasts, also found during inflammation and wound healing [14]. It is noteworthy that also in fibrotic diseases fibroblasts display a constitutively activated myofibroblast-like phenotype [15].

Anyway, CAFs can derive from the activation of resident fibroblasts or other precursor cells represented by bone marrow derived mesenchymal stem cells, epithelial cells, carcinoma cells, endothelial cells, pericytes, smooth muscle cells, adipocytes, fibrocytes, stellate cells in pancreas and liver, myoepithelial cells in breast, and pericryptal myofibroblasts of the gastrointestinal tract [11]. CAFs show high expression of alpha-smooth muscle actin (*α*-SMA), one of the most significant markers of fibroblasts activation and CAFs differentiation [11]. Furthermore, it is known that *α*-SMA expression is induced by overexpression of hypoxia-associated microRNA- (miR-) 210, which converts healthy fibroblasts into CAFs‐like cells [16].

However, *α*-SMA is not the only molecular marker useful to identify CAFs. In fact, fibroblast activation protein (FAP), fibroblast-specific protein 1 (FSP1), osteonectin, desmin, platelet-derived growth factor receptors (PDGFR) *α* or *β*, neuron-glial antigen-2 (NG2), periostin (POSTN), podoplanin (PDPN), tenascin-C (TNC), CD90/THY1, or discoidin domain-containing receptor 2 (DDR2) and the mesenchymal cell marker vimentin can be also considered CAF markers [11, 17, 18].

Furthermore, it is noteworthy that circulating breast CAFs in the peripheral blood of patients with metastatic breast cancer were characterized as FAP^+^/*α*-SMA^+^/Cytokeratin^−^/CD45^−^ [17].

In breast and pancreatic carcinoma, CAFs can represent up to 80% of the tumour mass, as a result of a widespread desmoplasia that generates mechanical forces activating fibroblasts [7]. Hence, desmoplasia and a lot of signalling pathways in tumours can induce differentiation of CAFs, which in turn promote tumour progression and metastasis through the secretion of growth factors and chemokines [7]. In addition, desmoplasia and proliferating tumour cells generate high consumption of oxygen and the growing tumour mass leads to progression of a hypoxic and acidic environment, towards which cancer cells must exhibit rapidly an adaptive response. The adaptation to hypoxia and hyponutrient conditions is sustained by the so-called “metabolic reprogramming”, i.e., a process in which changes of bioenergetics and biosynthesis occur both in cancer cells and CAFs [3, 7]. In this way, the remodelling of cancer metabolism ensures sufficient building blocks for biosynthesis and facilitates cancer cells to survive a harsh hypoxic and nutrient-deprived microenvironment by promoting tumour vascularization and bypassing cancer immunity [19].

Hence, in tumour microenvironment, CAFs seem to be enslaved by cancer cells to support their massive and uncontrolled proliferation and nutrients demand: in fact, CAFs directly fuel tumour cells by producing and exporting high energy metabolites, especially lactate, pyruvate, and ketone bodies, which are used by adjacent cancer cells [20]. Moreover, metabolic reprogramming is not utilized for biosynthesis in CAFs, whose proliferation rate is unexpectedly slower than that of NFs but is needed for cancer cells to generate energy, necessary to support cell division and to evade the checkpoints that would normally block cell proliferation under stressful condition [21].

## 3. Metabolic Signalling Pathways Associated with CAFs Differentiation

It is known that, in the absence of an abnormally activated tumour microenvironment, both genetic and epigenetic mutations in cancer cells are not sufficient to sustain cancer progression [8]. In fact, a necessary step for cancer initiation and progression is represented by CAFs differentiation, which can either occur at the early phase of cancer [9] or surprisingly precede the genetic alterations of epithelial cells, triggering the malignant transformation of adjacent cells [22]. The constitutive activation of tumour stroma leading to CAFs differentiation is associated with signalling pathways, modulated mainly by tumour cells, with autocrine loops [14, 23, 24] and with CAFs metabolic reprogramming found in many types of solid cancers, including breast, lung, prostate and gastric cancer, head and neck squamous cell carcinoma (HNSCC), and lymphomas [25, 26]. In particular, CAFs differentiation can be induced by tumour cell-derived transforming growth factor *β* (TGF-*β*), epidermal growth factor (EGF), PDGF*α*, PDGF*β*, basic fibroblast growth factor (bFGF, also known as FGF2), interleukin 6 (IL-6), and interleukin 1*β* (IL-1*β*) [11, 18].

Moreover, it is known reactive oxygen species (ROS) to regulate the metabolic reprogramming of both cancer cells and CAFs, supporting the adaptation to oxidative stress that triggers CAFs differentiation, tumorigenesis, and chemoresistance [4]. In the tumour microenvironment, cancer cells produce high levels of ROS deriving from mitochondrial dysfunction, upregulation of NADPH oxidase 1 (NOX-1) and NADPH oxidase 4 (NOX-4), and alterations of antioxidant enzymes [7]. In particular, the mitochondrial dysfunction is associated with a switch to aerobic glycolysis, known as “Warburg effect”, whcih is an early step of carcinogenesis, and can occur before the appearance of an important driver of the metabolic switch in tumour cells: the hypoxia [4, 7]. However, in cancer cells both “Warburg effect” and mitochondrial malfunctioning trigger an increase of lactate and ROS levels and a decrease of antioxidant molecules [7]. Therefore, ROS can initiate a cascade of intra- and intercellular events associated with metabolic switch in cancer [20] and CAFs formation [7]. In particular, the hydrogen peroxide (H_2_O_2_) produced by cancer cells induces in CAFs an oxidative stress, associated with the reduction of mitochondrial function and the increase of both glucose uptake and ROS levels, leading to CAFs differentiation [7]. Therefore, ROS generate a reactive microenvironment, where the energy needed for cancer cells proliferation is sustained by CAFs, whose activated phenotype is constantly maintained [7].

CAFs secrete higher levels of H_2_O_2_ compared with normal cells, suggesting that extracellular H_2_O_2_ could lead to stroma remodelling. Indeed, treatment of NFs with CAF-conditioned medium or exogenous H_2_O_2_ leads to the acquisition of an oxidative CAF-like state [27]. The higher H_2_O_2_ production by CAFs is due to an impaired TGF-*β* signalling leading to the suppression of the antioxidant enzyme glutathione peroxidase 1 (GPx1) [27].

TGF-*β* is a protein with a key role in CAFs differentiation, enabling the increase of fibroblasts ROS that modulate *α*-SMA expression [7]. In particular, it is known that TGF-*β* takes part in CAFs differentiation and metabolic regulation [28]. Indeed, TGF-*β* induces differentiation of prostate CAFs by triggering NOX-4 upregulation and ROS production [29]. Moreover, TGF-*β* triggers in fibroblasts increased oxidative stress, autophagy/mitophagy, aerobic glycolysis, and downregulation of caveolin-1 (Cav-1): these alterations can extend to surrounding fibroblasts and support cancer cell growth [30]. Additionally, TGF-*β* signalling pathway is also linked to the expression levels of some metabolic enzymes, such as isocitrate dehydrogenase 1 (IDH1). In particular, Jun Mi's group showed a novel regulation network between cell signalling pathway and cellular metabolism. TGF-*β* receptor (TGFBR)-IDH1-Cav-1 axis triggers TGF-*β* signalling in fibroblasts [28]. In turn, TGF-*β* signalling induces the downregulation of IDH1 expression and this downregulation enhances TGF-*β*-activated canonical Smad signalling. Despite IDH1 is an enzyme involved in the conversion of isocitrate to *α*-ketoglutarate (*α*-KG) in a NADP+-dependent manner, its depletion increases cellular *α*-KG levels that suppress Cav-1 expression. The Cav-1 downregulation inhibits TGFBR protein degradation and induces TGF-*β* signalling, supporting and increasing the effect of this autocrine loop. Furthermore, in murine xenograft tumour model, the protumorigenic effect of IDH1-knockdown fibroblasts is similar to CAFs one [28].

High levels of ROS, produced by cancer cells, induce oxidative stress in CAFs and lead to the production of autophagosomes that fuse with lysosomes, with a consequent mitochondria disruption and Cav-1 degradation [20, 31]. Loss of Cav-1, a marker of autophagy, glycolysis, and oxidative stress [31], is also sufficient to induce a constitutive activated phenotype in CAFs [32]. Ablation or mutation of Cav-1 is one of the features of fibroblasts in tumour tissues [33]. The downregulation of Cav-1 in CAFs results in higher ROS levels in cancer cells, which induce oxidative stress in CAFs in a positive feedback loop [20]. Furthermore, Cav-1 downregulation triggers a fibroblast shift toward catabolic metabolism and promotes the mitochondrial activity of adjacent cancer cells [34]. CAFs and cancer cells adopt these self-stimulating and cross-communicating pathways to maintain their protumorigenic potential. Additionally, the downregulation of Cav-1 in fibroblasts is associated with the induction of TGF-*β* signalling [30].

The increase of ROS levels in tumour environment also induces the proinflammatory transcriptional factor NF*κ*B activity in fibroblasts, leading to a CAF-like phenotype [35]. In fact, NFkB target gene cyclooxygenase-2 (COX-2) is found upregulated in several solid tumours and in CAFs [36, 37]. In addition, NFkB feeds the oxidative stress in CAFs causing a defect in ROS detoxification through Gpx inhibition [38].

Other factors involved in fibroblasts metabolic reprogramming linked to CAFs formation are represented by desmoplasia and hypoxia. In particular, desmoplasia of solid tumours generates mechanical forces converting fibroblasts and other precursor to myofibroblasts and originates a hypoxic and acidic microenvironment that impairs chemotherapeutic treatment [39]. Furthermore, hypoxic microenvironment of desmoplastic cancer tissues produces and maintains an oxidative stress condition, because hypoxia is linked to mitochondrial ROS production and glycolytic pathway [23].

Recent work studied the role of G-protein estrogen receptor (GPER) and hypoxia in CAFs differentiation [40]. GPER is a seven-transmembrane-associated estrogen receptor belonging to G-protein coupled receptors family, often upregulated in breast CAFs [41]. GPER modulates cell signalling pathways and promotes breast cancer proliferation, chemoresistance, and metastasis [42]. Furthermore, in breast CAFs GPER biological function is linked to stimulatory effects of estrogen and to regulation of the crosstalk between cancer cells and CAFs [41]. GPER expression in breast CAFs is associated with hypoxia-induced CAFs activation and breast cancer cell invasion [40]. In fact, GPER knockdown abrogates hypoxia driven CAFs formation, inhibits breast cancer cell invasion induced by CAFs conditioned medium, and abolishes hypoxia-activated connective tissue growth factor (CTGF), vascular endothelial growth factor (VEGF), and IL-6 secretion in CAFs [40].

It is known that exosomes secreted by cancer cells are linked to CAFs differentiation [43]. In particular, exosomes from prostate cancer cells contain high levels of IL-6 that modulates, together with other signalling molecules, microenvironment remodelling and CAFs transdifferentiation [44]. Moreover, increased IL-6 expression has been also detected in breast and ovarian CAFs [37]. Additionally, it is noteworthy that IL-6 links CAFs inflammation to the enhancement of glycolysis: this process could be associated with expression of the glycolytic enzymes hexokinase 2 (HK2) and 6-phosphofructo-2-kinase/fructose-2,6-bisphosphatase-3 (PFKFB3) induced by IL-6 [43, 45].

## 4. CAFs Metabolic Reprogramming and Cancer Crosstalk

**Figure 1:**
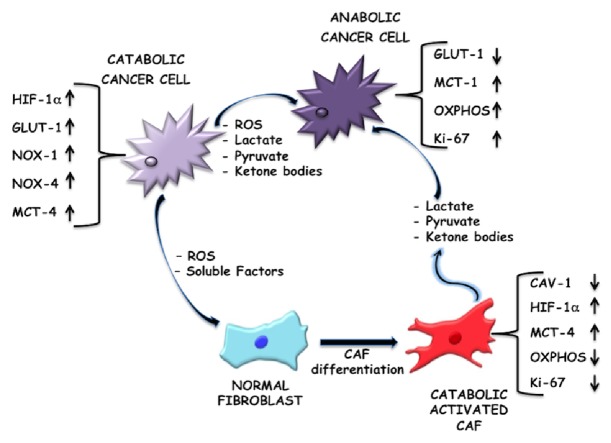
Metabolic reprogramming of tumour microenvironment, a three-compartment model. Tumour growth and progression are sustained by a metabolic interplay between catabolic tumour cells, normal fibroblasts, and catabolic activated CAFs that contribute to the anabolic reprogramming of cancer cells. This crosstalk is mediated by ROS, soluble factors, energy-rich fuels, and catabolite transporters, such as monocarboxylate transporter 1 (MCT-1), monocarboxylate transporter 4 (MCT-4), and glucose transporter protein (GLUT-1). In particular, mitochondrial dysfunction in catabolic cancer cells is associated with glycolysis switch (“Warburg effect”). These catabolic tumour cells show an increase of glucose uptake, upregulation of NOX-1 and NOX-4, and high level both of ROS and energy-rich fuels extrusion. The differentiation of normal fibroblasts into activated CAFs is ROS modulated. ROS, produced by catabolic cancer cells, upregulate HIF-1*α* whose levels are also increased by the loss of caveolin-1 (Cav-1). These events are involved in CAFs glycolytic switch. Hence, CAFs show a catabolic phenotype characterized by an inhibition of OXPHOS, a reduction of proliferation marker Ki-67, and release of energy-rich fuels. These molecules, represented by lactate, pyruvate, ketone bodies, etc., feed cancer cells that acquire an anabolic phenotype, where the high request of ATP is satisfied by an efficient mitochondrial OXPHOS.

The most of cancer cells do not rely primarily on mitochondrial oxidative phosphorylation (OXPHOS) but can produce energy, needed for cellular processes, via the conversion of glucose into lactate, despite the presence of sufficient oxygen [5, 46]. Cancer cells in the solid tumour core have to assemble a compensatory environment around them, in which CAFs become their metabolic slaves providing crucial metabolic intermediates for adenosine triphosphate (ATP) synthesis [32, 47]. Hence, CAFs undergo metabolic reprogramming switching towards a more glycolytic phenotype, whereas the cancer cells rely more on their mitochondrial routes of energy production via OXPHOS. In fact, it has been shown that MCF7 breast cancer cells generate 80% of their ATP through mitochondrial respiration [48]. Additionally, inhibiting glycolysis in neoplastic cells restores mitochondrial OXPHOS, demonstrating that oxidative metabolism remains functional in most glycolytic cancer cells [4]. These findings are the antithesis of the classical Warburg's hypothesis which assumes the tumour cells to be highly glycolytic in nature and to have an impaired mitochondrial activity. Hence, this alternative idea of aerobic glycolysis in CAFs and not in cancer cells, as previously thought, supporting the oxidative tumour mass was termed as the “reverse Warburg hypothesis” [49–51]. “The reverse Warburg effect” can be explained by a two-compartment tumour metabolism model, in which anabolic cancer cells and catabolic CAFs are metabolically coupled. Briefly, catabolic CAFs, through aerobic glycolysis, generate higher levels of energy-rich fuels, to feed mitochondrial OXPHOS in the adjacent anabolic cancer cells [20]. This two-compartment model has been further amplified in a three-compartment tumour metabolism model, in which catabolic stromal and catabolic cancer cells are metabolically coupled to anabolic cancer cells, via catabolite transporters (MCTs) (Figure 1). This metabolite compartment asymmetry further shows the complexity of tumour ecosystem, demonstrating that cells with different metabolic phenotype coexist and act together to sustain tumour growth and diffusion (Figure 1) [31].

Glycolysis-related enzymes, such as HK2 and 6-phosphofructokinase liver type (PFKL), are considerably upregulated in CAFs, substantiating their glycolytic nature [32, 43, 52]. In particular, HK2 is a pivotal glycolytic enzyme that is overexpressed in tumours and contributes to “Warburg effect” [53]. In a CAF model, HK2 protein levels increase during CAFs differentiation induced by TGF-*β*1. Furthermore, HK2 upregulates p27 protein expression through its downstream metabolite *α*-KG. In turn p27 inhibits cyclin-dependent kinase 2 (CDK2) and activates the G1/S checkpoint. This regulatory mechanism connects glycolysis to cell cycle control: in fact, HK2 enzyme regulates both glycolysis and a cell cycle checkpoint [53].

The high rate of glycolysis is believed to be one of the driving forces behind the supportive role of CAFs in tumour growth. However, up to now, the molecular mechanisms responsible for this change achieved in CAFs are not fully understood and defined. A large number of possible mechanisms has been proposed and explored to explain the metabolic reprogramming associated with the upregulation of glycolysis in CAFs. CAFs are reprogrammed by contact with cancer cells toward a glycolytic phenotype, increasing their glucose upload and their delivery of lactate and pyruvate, the end products of glycolysis [47, 54]. In particular, the entry of glucose into the cells is allowed by enhanced expression of GLUT-1. It is known that oncogene like cMyc enhances the metabolic flux and glucose uptake by increasing the lactate dehydrogenase-A (LDH-A) but also GLUT-1 expression [55]. Moreover, downregulation of miR-186 increases GLUT-1 protein level during CAFs formation. Therefore, miR-186 modulates glycolysis through GLUT-1 [56].

The extrusion of lactic acid, instead, is assured by monocarboxylate transporter-4 (MCT-4). Specifically, lactate is always extruded by MCT-4 in association with the H^+^, leading to the acidification of tumour microenvironment [47]. Increased acidity causes activation of matrix metalloproteinase-9 (MMP-9) and enhances epithelial mesenchymal transition (EMT) of neighboring cancer cells, positively affecting tumour progression [57]. Hence, increased glycolysis, showed by excessive lactate production, leads to upregulation of MCT-4 in CAFs such as observed in breast and bladder CAFs [58].

On the other hand, monocarboxylate transporter 1 (MCT-1) is the transporter responsible for lactate influx into cancer cells, as can be observed in the osteosarcoma cells [59, 60]. Lactate is efficiently exploited by cancer cells themselves both to obtain energy and biomolecules through enhanced anabolism and to fuel OXPHOS. This evidence further strengthens the concept of metabolic reprogramming in tumour microenvironment.

As mentioned above, extracellular acidification is an important feature for tumour progression and it has been mainly correlated with metabolic reprogramming of tumour cells toward Warburg metabolism [61]. Carbonic anhydrases (CAs) are a family of zinc metalloenzymes that rapidly catalyse the hydration of carbon dioxide, producing bicarbonate and protons. At least thirteen human active isoenzymes belong to this family and, in particular, CA IX is a transmembrane enzyme endowed with an extracellular membrane-bound catalytic domain that contributes to acidification of the outer microenvironment. Within tumours CA IX is mainly distributed in perinecrotic areas, likely due to its acknowledged regulation by hypoxia through hypoxia-inducible factor 1 *α* (HIF-1*α*). Fiaschi et al. showed an upregulation of CA IX in CAFs upon contact with prostate carcinoma cells, concurring to extracellular acidification [61].

It is known that GPER estrogen receptor is able to modulate CAFs metabolic reprogramming. In particular, Yu et al. demonstrated that GPER is transferred from the nucleus to the cytoplasm of estrogen-stimulated breast CAFs only upon a direct contact with breast tumour cells. The cytoplasmic GPER, through the activation of GPER/cAMP/PKA/CREB signalling, induces the energy metabolism switch of CAFs towards a “Warburg-like state”, supporting the critical role of CAFs and tumour cells crosstalk in the metabolic reprogramming and in breast cancer progression [25].

Another process associated with the metabolic remodelling in CAFs implicates tricarboxylic acid (TCA) cycle downregulation. Zhang et al. identified Krebs cycle enzyme isocitrate dehydrogenase 3*α* (IDH3*α*) downregulation as a critical marker for switching energy metabolism from OXPHOS to glycolysis in TGF-*β*1/PDGF-induced CAFs [52]. Moreover, miR-424 downregulates IDH3*α* whose overexpression prevents the differentiation of NFs to CAFs. In primary fibroblasts, with IDH3*α* knockdown, glucose uptake and lactate production are increased, whereas oxygen consumption is decreased [52]. In contrast, IDH3*α* overexpression not only reduces the basal level of TGF-*β*-stimulated glucose uptake but also inhibits TGF-*β*-induced lactate production with increased basal oxygen consumption. Furthermore, downregulation of IDH3*α* decreases the effective level of *α*-KG by reducing the ratio of *α*-KG to fumarate and succinate, required for prolyl hydroxylase domain-containing protein 2 (PHD2) activity. PHD2 is a HIF-1 downregulator and its inhibition allows HIF-1*α* protein stabilization in the cytosol [62, 63]. Under normoxic condition, HIF-1 is destined for ubiquitination and degradation that instead is prevented in oxygen deprived conditions leading to its accumulation in the cytosol [64]. The stabilization of HIF-1*α* and its subsequent nuclear translocation is considered as one of the pivotal events inside the hypoxic solid tumour core. HIF-1*α* is associated with the upregulation of about 100 genes, several of which are directly related to the glycolytic pathway [65]. Hence, the accumulation of HIF-1*α*, in turn, promotes glycolysis by increasing the uptake of glucose and inhibiting OXPHOS by upregulating NADH dehydrogenase ubiquinone 1 alpha subcomplex, 4-like 2, (NDUFA4L2) a negative regulator of mitochondrial complex 1 [52]. In addition, a previous study showed that HIF-1*α* upregulates NDUFA4L2 expression during hypoxia in mouse embryonic fibroblasts (MEFs) and tumour cells [66]. Taken together, these data indicate that IDH3*α* downregulation upregulates HIF-1*α* and NDUFA4L2, which in turn promotes glycolysis and inhibits OXPHOS, respectively, providing an insight into the initiation of “Warburg-like effect” in CAFs.

HIF-1 is not only activated upon low O_2_ concentration but also under normoxic conditions. HIF-1*α* stabilization is brought about by a loss of Cav-1, leading to an induction of oxidative stress in CAFs creating a pseudo-hypoxic state [67]. A proteomic analysis of Cav-1-deficient fibroblasts in human breast cancer tissues revealed an increased transcription level of glycolytic enzymes under normoxic conditions [32]. Subsequent study showed that loss of Cav-1 in mesenchymal stromal cells leads to increased aerobic glycolysis via activation of HIF-1 and NFkB favouring tumour growth [67, 68]. In support of this hypothesis, a study performed in a xenograft model evidenced that the HIF-1*α*-dependent activation of autophagy in stromal cells greatly enhances the tumorigenicity of MDA-MB-231 breast cancer cells [34]. Moreover, HIF-1*α* expression is shown to be directly associated with the main exporter of lactate in CAFs, MCT-4 [31].

As shown by Balliet and colleagues, the downregulation of mitochondrial transcription factor A (TFAM) in fibroblasts is linked to Cav-1 dysregulation, with a consequent induction of oxidative stress, mitochondrial dysfunction, and aerobic glycolysis in the tumour microenvironment. TFAM deficient fibroblasts produce more H_2_O_2_ and L-lactate and are sufficient to promote tumour growth [69]. Finally, the Cav-1-knockout fibroblasts metabolically cooperate with cancer cells by enhancing lactate production for mitochondrial respiration in anabolic cancer cells [32] Taken as a whole, the loss of stromal Cav-1 is very important in the metabolic reprogramming of CAFs.

For the metabolic crosstalk between CAFs and cancer cells, the HIF-1-driven transcriptional activity is important and can also be mediated by activation of sirtuin1 (SIRT1) signalling that ensures deacetylation of peroxisome proliferator activated receptor gamma coactivator 1*α* (PGC1-*α*) or by mitochondrial deacetylase SIRT3 downregulation that increases the level of inactive superoxide dismutase 2 (SOD2) acetylated [47, 54]. These events lead to increased mitochondrial function associated with overproduction of ROS and subsequent functional regulation of pyruvate kinase M2 (PKM2). The expression of PKM2 is also induced in Cav-1-knockdown fibroblasts and is a sufficient condition to trigger aerobic glycolysis [34]. In cancer cells after contact with their stromal CAFs, PKM2 acts by regulating OXPHOS addiction, instead of the classical Warburg glycolytic metabolism. Indeed, upon CAFs contact PKM2 becomes oxidized by ROS delivered by hyperactive mitochondria, as well as tyrosine phosphorylated by activated Src kinase. PKM2 migrates into the nucleus and recruits both HIF-1 and associate embryo-chondrocyte expressed gene-1 (DEC1), thereby repressing expression of miR-205, driving a pleiotropic transcriptional response leading to metabolic reprogramming toward OXPHOS and enhancing survival and EMT [47].

In addition to lactate, previous studies have shown that CAFs also increase the production of other nutrients like glutamine and ketone bodies, which emerge as possible fuel sources for anabolic metabolism or OXPHOS, utilized by tumour cells in support of their growth [31, 70].

A vast array of studies indicates that tumour cells induce a metabolic overdrive in CAFs, also almost to the point of self-destruction of cell's own organelles and protein molecules, inducing autophagy and mitophagy [9, 71]. These are processes by which CAFs recycle the important biomolecules and metabolic precursors to generate lactate and pyruvate via aerobic glycolysis. These two metabolites are continually channelled towards the nearby tumour cells, which utilize them to manufacture additional ATP molecules via oxidative mitochondrial metabolic pathways [43, 72].

Previous studies have demonstrated that loss of Cav-1 in stromal cells enhances the transcription of TGF-*β* target genes, such as CTGF. It is known that CTGF overexpression in fibroblasts induces an autophagy/mitophagy program, only downstream from a loss of stromal Cav-1 [34]. CAFs adopt this self-destructive mechanism to create a nutrient-rich microenvironment by release of lactate, ketone bodies, and glutamine, metabolically supporting cancer growth [73].

Furthermore, Santi et al. showed that CAFs, using microvesicles (MVs) as cargo, are also able to transfer a large amount of proteins and lipids to neighboring cancer cells, thereby contributing to sustain the high proliferation rate of tumour cells [74]. Since several transferred proteins are metabolic enzymes, MVs have an important role in metabolic reprogramming of cancer cells due to CAFs contact.

Additionally, also CAF-derived exosomes are able to induce a metabolic reprogramming in cancer cells after their uptake [75]. Zhao et al. demonstrated that CAF-derived exosomes contain intact metabolites, like amino acids, lipids, and TCA-cycle intermediates that are vehiculated in cancer cells to support their growth [76].

To sum up, regarding the exploration and the function of the mechanisms underlying this metabolic reprogramming, it appears that the metabolic behaviour sustains growth of cancer cells at the clear expenses of the stromal counterpart with a proliferation rate of CAFs lower than NFs.

Furthermore, the aerobic glycolysis and concomitant increase in glucose uptake make the positron emission tomography (PET) an imaging technology, which uses glucose analog tracer for tumour diagnosis, able to detect glucose consumption in stroma rather than strictly in cancer cells [4]. In fact, it is noteworthy that in the tumour mass CAFs have the largest increases in glucose uptake. Martinez-Outschoorn et al. suggested that the PET scanning with 2-[18F]-2-deoxy-D-glucose (18F-FDG), currently used to measure the extent of fibrosis in a number of human diseases, such as pulmonary fibrosis, postsurgical scars, and arthritis, may specifically detect the tumour stroma in cancer patients, by visualizing the glucose uptake and thereby the “Warburg effect” in CAFs, rather than in cancer cells [77].

## 5. Therapeutic Responses to CAFs Metabolic Reprogramming

The metabolic symbiosis between cancer cells and stromal cells is increasingly recognized as the main driver of tumour progression, metastasis, and therapeutic failures.

The mutagenic and oxidative stress, propagated from cancer cells to CAFs, and vice versa, generates a very unstable and lethal microenvironment, in which cancer cells exploit metabolically CAFs to support their own survival and growth. In fact, during metabolic symbiosis, CAFs help cancer cells to overcome cellular and pharmacological stress reducing apoptosis [78] and increasing mitochondrial activity in cancer cells [51]. Indeed, cancer cells that acquire drug resistance are characterized by increased mitochondrial mass, OXPHOS activity, and antioxidant capacity. Initially, anticancer drugs reduce the tumour mass, by damaging or killing the major population of cancer cells. Unfortunately, this initial cancer regression often precedes the appearance of new and more vigorous tumours due to the surviving residual cancer cells that resist the pharmacological stress via mitochondrial adaptation [79]. Martinez-Outschoorn et al. demonstrated that two potent mitochondrial “poisons”, namely, metformin and arsenic trioxide (ATO), are able to resensitize breast cancer cells, whose tamoxifen resistance has been induced by CAFs [78]. Moreover, metformin, usually used in the treatment of diabetes, is currently undergoing phase 2/3 clinical trials as adjuvant therapy in several cancer types, for its capacity to restore Cav-1 expression in CAFs [80]. In fact, metformin, through AMP activated protein kinase (AMPK) induction, inhibits autophagy, which is the process involved in Cav-1 degradation. In addition, metformin is currently utilized in phase 1 study in combination with a specific inhibitor of autophagy, called temsirolimus, to treat patients with aggressive B-cell lymphoma [81].

Furthermore, in the last few years, chloroquine underwent a clinical trial known as Preventing Invasive Breast Neoplasia with chloroquine (PINC) because of its ability to rescue the expression of Cav-1 in CAFs via the inhibition of autophagy [82].

However, metabolic symbiosis between CAFs and cancer cells can represent an adaptive response to cancer therapy, which results in drug resistance. In mouse models of breast tumour, cancer cells overcome nintedanib treatment shifting towards a hyperglycolytic metabolism and inducing the overexpression of MCT-4 [83], a well-known marker of oxidative stress in CAFs [58]. The genetic ablation of MCT-4 expression is sufficient to overcome therapy resistance and enhance the antitumour effect of nintedanib [83]. Therefore, MCTs offer a great potential for developing new anticancer therapies: in support of this notion, several studies have demonstrated that the genetic disruption of MCT-1 or MCT-4 blocks breast tumour growth [84] and sensitizes cancer cells to treatment with phenformin, an inhibitor of mitochondrial complex 1 [85].

Moreover, growing evidence suggests that the cytoplasmic stromal GPER, involved in the aerobic glycolysis switch in CAFs, as already described in this review, is also implicated in the development of multiple drug resistance to classical clinical drugs, such as tamoxifen, herceptin, and epirubicin [25]. In fact, drug-resistant tumours show high levels of the cytoplasmic stromal GPER and extremely increased aerobic glycolysis. This is confirmed by 18F-FDG PET/CT analysis showing an important association between the GPER/cAMP/PKA/CREB pathway of stromal fibroblasts and 18F-FDG uptake in primary or drug-resistant tumours [25]. Hence, the cytoplasmic GPER in CAFs may represent another promising target for cancer therapy to rescue the drug sensitivity in patients with breast cancer [25].

Furthermore, the increasing knowledge about the capacity of ROS production and oxidative stress to induce in CAFs an inflammatory phenotype and tumour stroma metabolic coupling [86] supports the idea that treatment with antioxidants and/or anti-inflammatories may allow the metabolic separation of cancer cells from CAFs, leading to cancer cells death and consequently tumour regression. Martinez-Outschoorn and colleagues demonstrated that treatment with antioxidants, such as N-acetyl-cysteine (NAC), metformin, and quercetin or nitric oxide (NO) inhibitors, like L-NAME, is useful to reverse CAFs phenotypes, rescuing Cav-1 expression in fibroblasts [87]. The antineoplastic activity of metformin is also associated with its ability to reduce endogenous ROS production, oxidative stress, and related DNA damage and mutations [88]. In addition, the antioxidants NAC, quercetin, metformin, and chloroquine dramatically reduce MCT-4 expression in CAFs [89, 90]. In fact, Monti et al. demonstrated in a clinical trial that NAC, reducing MCT-4 expression in the tumour stroma of cancer patients, decreases carcinoma cell proliferation rates in women with stages 0 and I breast cancer. This pilot clinical trial showed NAC effectiveness and safety in breast cancer treatment [91]. Moreover, NAC in combination with topotecan underwent a phase 2 clinical trial in ovarian cancer patients, based on their role in the regulation of Cav-1, MCT-4, and HIF-1*α* expression [20].

Accumulating evidence suggests that nonsteroidal anti-inflammatory drugs (NSAIDs), such as aspirin, celecoxib, and diclofenac, are associated with a decreased risk of colorectal, lung carcinomas, and other tumours [92–94]. In particular, the anticancer effect of aspirin may be explained by its ability to affect the metabolism in cancer. In fact, aspirin both triggers the suppression of de novo lipogenesis in prostate and lung cancers [95] and induces posttranslational modifications of enzymes of the glycolytic pathway and mitochondrial proteins, leading to a change in their function [96].

LDH-A, an enzyme involved in the conversion of pyruvate to lactate, is highly expressed in Cav-1 (−/−) null stromal cells [97]. For this reason, a selective suppressor of LDH-A, named FX11, able to reduce the progression of human lymphoma and pancreatic cancer xenografts [98] was studied by National Cancer Institute's Experimental Therapeutics Program (NExT) [99].

As described in this review, low pH in tumours is the consequence of high metabolic activities and an important driver of tumour progression and aggressiveness. For this reason, many drugs targeting proton transporters have been suggested as anticancer drugs. In particular, a CA IX inhibitor, indisulam, underwent a phase 2 clinical trial for the treatment of melanoma, lung, pancreatic, and metastatic breast cancers, although no significant efficacy was observed in a phase 2 clinical trial on non-small cell lung cancer [100].

In an orthotopic mouse model for ovarian carcinoma, the simultaneous depletion of glutamine synthetase (GS), upregulated in CAFs during metabolic coupling, and glutaminase (GLS), expressed in cancer cells, results in a greater reduction of tumour growth and metastasis with respect to monotherapy. The concomitant use of GS and GLS inhibitors may represent a novel and lethal approach to target tumours and disrupt the metabolic crosstalk between stromal and cancer cells [101]. Hence, the inhibition of enzymes associated with energy-rich fuels overproduction in CAFs can be considered as new promising therapeutic targets in cancer patients treatment [97]. Indeed, the reduction of the bioenergetic support of CAFs in the tumour mass may induce starvation and/or death of cancer cells, leading to cancer regression [86].

HK2 is considered an important anticancer drug target. In fact, HK2 inhibits mitochondrial apoptosis by direct insertion in the mitochondrial outer membrane and induces drug resistance. Due to its contribution in regulating apoptosis and cellular bioenergetics, HK2 inhibitors have been developed [75]. In particular, as discussed by Gatenby and Gillies the HK2 inhibitor 3-bromopyruvate (3-BP) is able to reduce ATP reserves and thereby reverse chemoresistance [102]. Moreover, in order to reduce the adverse effects of 3-BP, due to its nonspecific delivery and distribution to healthy organs, 3-BP was encapsulated into a liposomal nanocarrier (T-Lipo-3-BP) and specifically delivered to the tumour mass after systemic administration in a mouse tumour model. Zhang et al. demonstrated that T-Lipo-3-BP nanoparticles represent a safe and efficient controlled release system: this novel therapeutic approach abolishes the severe side effects, such as the hepatotoxicity of 3-BP, and suppresses tumour growth [103].

Unfortunately, one of the most side effects of cancer chemotherapy is represented by the growth of a second primary tumour that does not derive from metastatic growth [104]. This process could be associated with activation of stromal fibroblasts induced by chemotherapy drugs. In particular, recent* in vitro* study showed that treatment of stromal fibroblasts with commonly used anticancer drugs induces CAFs differentiation [104]. Furthermore, upon treatment, stromal fibroblasts trigger stemness, antioxidant, and immune response in breast cancer cells. Hence, new and specific antistromal therapies must be necessarily added to the traditional antitumour drugs to intensify the fight against cancer (Table 1).

## 6. Conclusions

Tumour initiation and progression need metabolic reprogramming of tumour microenvironment. Additionally, solid tumours can be considered as metabolically heterogeneous diseases where several cell types and energetic pathways coexist and collaborate to assure the growth and progression of pathology. In this biological scenario, CAFs could represent the main cell type regulating the homeostasis and crosstalk within cancer tissues. This hypothesis is supported by the heterogeneity of fibroblasts cell population associated with functional diversity and by capability of activated fibroblasts of modulating inflammation process [105–107] that is one of the main leading causes of cancer progression [108].

The importance of CAFs in tumour pathogenesis is further strengthened by their involvement in cancer initiation, metastasis, angiogenesis, lymphangiogenesis, metabolic reprogramming, and therapy resistance [9].

Moreover, the presence of breast CAFs detected in the peripheral blood of patients with metastatic breast cancer [10] confirms the dramatic adaptability of this cell type.

Therefore, from our point of view, the metabolic slavery of CAFs within tumour microenvironment represents a central topic of the oncological research. In particular, the development of strategies committed to inactivate CAFs myofibroblastic phenotype [109] and to disconnect the metabolic crosstalk between CAFs and cancer cells could contribute to eliminate protumorigenic activity of CAFs in cancer network.

## Figures and Tables

**Table 1:** List of several compounds targeting CAFs metabolism.

Compound	Mechanism of action	Pathway target	Refs
Metformin	↑ Cav-1; ↓ MCT-4	Oxidative stress Autophagy Lactate transporter	[80, 87, 89, 90]
Quercetin	↑ Cav-1; ↓ MCT-4	Oxidative stress Lactate transporter	[87, 89]
Chloroquine	↑ Cav-1; ↓ MCT-4	Autophagy Lactate transporter	[82, 89]
NAC	↑ Cav-1; ↓ MCT-4	Oxidative stress Lactate transporter	[20, 89–91]
L-NAME	↑ Cav-1	Oxidative stress Mitochondrial activity	[87]
FX11	↓ LDH-A	Lactate production	[98, 99]
Indisulam	↓ CA IX	Microenvironment acidification	[100]
3-BP	↓ HK-2	Glycolysis	[102]
T-Lipo-3-BP	↓ HK-2	Glycolysis	[103]
